# Spatial Trueness Evaluation of 3D-Printed Dental Model Made of Photopolymer Resin: Use of Special Structurized Dental Model

**DOI:** 10.3390/polym16081083

**Published:** 2024-04-12

**Authors:** Aonan Wen, Ning Xiao, Yujia Zhu, Zixiang Gao, Qingzhao Qin, Shenyao Shan, Wenbo Li, Yuchun Sun, Yong Wang, Yijiao Zhao

**Affiliations:** 1 Center of Digital Dentistry/Department of Prosthodontics, Peking University School and Hospital of Stomatology & National Center for Stomatology & National Clinical Research Center for Oral Diseases & National Engineering Research Center of Oral Biomaterials and Digital Medical Devices & Beijing Key Laboratory of Digital Stomatology & NHC Key Laboratory of Digital Stomatology, Beijing 100081, China; 2311110627@stu.pku.edu.cn (A.W.); xn1995@pku.edu.cn (N.X.); 2111110537@pku.edu.cn (Y.Z.); 2211210502@pku.edu.cn (Q.Q.); kqsyc@bjmu.edu.cn (Y.S.); 2 Department of Stomatology, Peking Union Medical College Hospital, Chinese Academy of Medical Sciences and Peking Union Medical College, Beijing 100730, China; 3 Institute of Medical Technology, Peking University Health Science Center, Beijing 100191, China; gzx123@pku.edu.cn (Z.G.); shenyaoshan@stu.pku.edu.cn (S.S.); liwenb@pku.edu.cn (W.L.)

**Keywords:** dentistry, dental model, stereolithography, photo-curing, rapid prototyping, linear measurement, 3D analysis, accuracy, trueness

## Abstract

(1) Background: Various 3D printers are available for dental practice; however, a comprehensive accuracy evaluation method to effectively guide practitioners is lacking. This in vitro study aimed to propose an optimized method to evaluate the spatial trueness of a 3D-printed dental model made of photopolymer resin based on a special structurized dental model, and provide the preliminary evaluation results of six 3D printers. (2) Methods: A structurized dental model comprising several geometrical configurations was designed based on dental crown and arch measurement data reported in previous studies. Ninety-six feature sizes can be directly measured on this original model with minimized manual measurement errors. Six types of photo-curing 3D printers, including Objet30 Pro using the Polyjet technique, Projet 3510 HD Plus using the Multijet technique, Perfactory DDP and DLP 800d using the DLP technique, Form2 and Form3 using the SLA technique, and each printer’s respective 3D-printable dental model materials, were used to fabricate one set of physical models each. Regarding the feature sizes of the simulated dental crowns and dental arches, linear measurements were recorded. The scanned digital models were compared with the design data, and 3D form errors (including overall 3D deviation; flatness, parallelism, and perpendicularity errors) were measured. (3) Results: The lowest overall 3D deviation, flatness, parallelism, and perpendicularity errors were noted for the models printed using the Objet30 Pro (overall value: 45 μm), Form3 (0.061 ± 0.019 mm), Objet30 Pro (0.138 ± 0.068°), and Projet 3510 HD Plus (0.095 ± 0.070°), respectively. In color difference maps, different deformation patterns were observed in the printed models. The feature size proved most accurate for the Objet30 Pro fabricated models (occlusal plane error: 0.02 ± 0.36%, occlusogingival direction error: −0.06 ± 0.09%). (4) Conclusions: The authors investigated a novel evaluation approach for the spatial trueness of a 3D-printed dental model made of photopolymer resin based on a structurized dental model. This method can objectively and comprehensively evaluate the spatial trueness of 3D-printed dental models and has a good repeatability and generalizability.

## 1. Introduction

Unlike other medical disciplines, dentistry highly emphasizes individualized treatment and personalized production, providing ample opportunity for the application of three-dimensional (3D) printing. As an advanced additive manufacturing technology integrating the latest achievements in mechanical engineering, computer numerical controlled manufacturing, laser technology, and materials science, photo-curing 3D printing has many advantages over traditional methods in terms of price, speed, reliability, and cost-in-use [1]. Photo-curing 3D printing techniques have already been widely applied in fabricating various products such as dental models, surgical guides, and dental prostheses. Typical photo-curing techniques are stereolithography (SLA), digital light processing (DLP), and photopolymer jetting (PPJ), which mainly include the Polyjet and Multijet techniques [2].

In modern dentistry scenarios, 3D-printed dental models have become increasingly valued by clinical practitioners [3], and 3D-printed dental models have been widely used in orthodontics, prosthodontics, and other disciplines. Although traditional plaster models meet current clinical demands, these models are fragile and require a great amount of storage space [4]. With intraoral and extraoral scanning devices emerging, easy access to digital dental models provides a perfect solution to these problems and, moreover, it revolutionizes the treatment process by virtue of dedicated digital software. For the purpose of converting digitized dental models into physical models, which is still indispensable in many clinical situations, photo-curing 3D printing has become the most frequent manufacturing method used. A key requirement for 3D-printed dental models is accurately replicating the surface morphology of a patient’s teeth and surrounding soft tissues [5,6]. However, polymerization-induced shrinkage can cause internal stress in printed dental models, which will result in strain deformation and, subsequently, in poor dimensional accuracy [7,8]. In addition, the morphological structure design and filling percentage of the model also have a certain influence on its printing accuracy [9,10]. Therefore, many efforts have been devoted to evaluating the accuracy of 3D-printed dental models in the past decade [11]. Even if lots of evaluation results reported are available, whether the accuracy of 3D-printed dental models outperforms that of plaster ones or milled ones is still controversial [12,13,14,15,16,17,18,19,20,21].

The term accuracy consists of two criteria, trueness and precision, according to the ISO standard (ISO 5725-1:2003) [22]. Trueness deals with systematic errors of measurement, referring to the closeness between the average test value and an accepted reference value [23]. From the clinical perspective, trueness is more considered by practitioners. To date, linear measurement is a widely adopted approach to quantify the trueness of 3D-printed models and, in the interim, determining the linear measurement landmark is inevitable [15,17,24,25,26,27,28,29,30,31,32,33,34,35]. Nevertheless, the irregular surfaces of dental models, having anomalistic pits and fissures, complicate the standardization of landmark identification, which can introduce unpredictable manual errors [17]. According to Saleh et al., the error of repetitive manual point selection on a dental model’s surface can reach 180 μm, in excess of the theoretical accuracy of current high-accuracy 3D printers [27]. To address this issue, hemispherical markers designed for linear measurement are used in some studies, so as to achieve a more reliable measurement [30]. But additional scanning and registration steps are also required to locate these reference markers. Tahayeri et al. designed a geometric reference model to evaluate the trueness of a 3D-printed multi-unit prosthesis by using a digital caliper to perform measurements directly on the physical printed model, avoiding the unnecessary model scanning [36]. Although the reference model with certain morphological characteristics used in this study has improved the reliability of linear measurements, the model is too simplified and differs markedly from the size and shape of real clinical situations, which leads to poor reference values. Using a geometric reference model is a common approach to evaluate the trueness of 3D-printed parts [37]. However, the amount of deformation caused by linear shrinkage in the stereolithography process tends to increase with the part’s size [8]. It is essential to design a geometric model with a size and shape simulating real dental arch.

In the present study, a set of structurized dental models with full dentition characteristics were designed, simulating a clinical dental model with 96 redefined feature sizes that can be easily measured by a hand-held digital caliper, a readily available tool. With the aid of an optical scanner and 3D analysis software, multi-dimensional evaluation results of 3D form trueness can also be gained.

This study aimed to evaluate the spatial trueness of photo-curing 3D-printed dental models, using a novel structurized model introduced previously [38] to provide objective assessment results of six types of representative printers. The hypotheses of this study were as follows: the method proposed in this paper can objectively and comprehensively evaluate the spatial trueness of 3D-printed dental models, and there are no differences in the spatial trueness among the six different photo-curing 3D printers.

## 2. Materials and Methods

### 2.1. Establishment of the Structurized Dental Model

**Figure 1:**
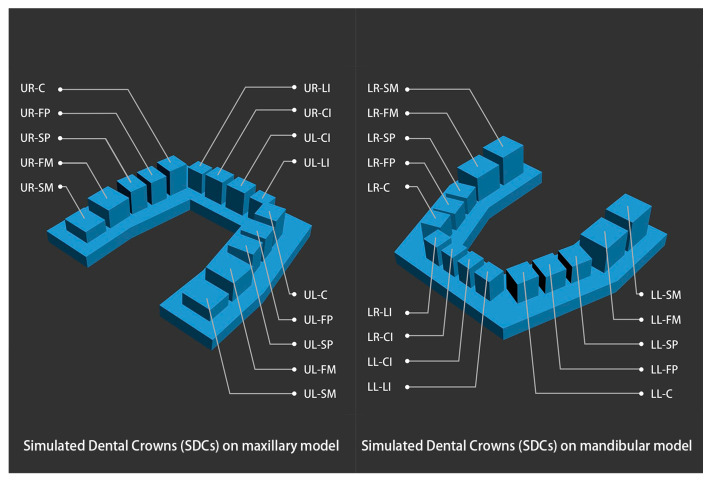
The simulated dental crowns (SDCs) of the structurized dental models. UL, upper left. UR, upper right. LL, lower left. LR, lower right. CI, central incisor. LI, lateral incisor. C, canine. FP, first premolar. SP, second premolar. FM, first molar. SM, second molar.

A set of structurized dental models, including a maxillary and mandibular models, was designed in 3ds Max software version 2018 (Autodesk, San Rafael, CA, USA). These virtual structurized models were based on dental crown and arch measurement data from the Chinese population, reported in previous studies [39,40,41,42], and comprised several simple geometrical configurations, which simplified the landmark selection step when performing linear measurements. Simulated dental crowns (SDCs) were cuboids used to simulate the dimensions of real dental crowns. A horseshoe-shaped base was thought to provide a better view of the 3D printer’s performance in terms of reduction in the transverse dimension and is also the mainstream design of 3D-printed dental models [28]. The dimensions of the model are shown in Table 1 and Figure 1.

### 2.2. Fabrication of the 3D-Printed Structurized Dental Model

**Figure 2:**
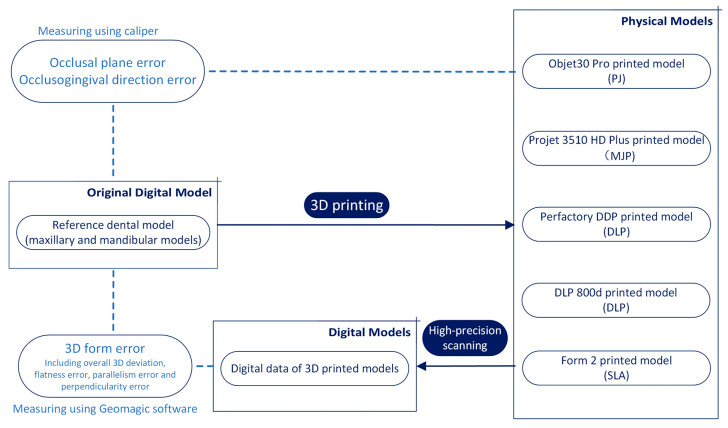
Schematic figure illustrating the study workflow.

Figure 2 illustrates the design of the study.

The 3ds Max software 2018 output the 3D structurized model as standard triangulated language (STL) data for the subsequent 3D printing and 3D evaluation steps. In this study, six types of photo-curing 3D printers were selected, which cover the above three mainstream printing technologies and had sufficient technical representation. They have been widely used in the field of stomatology research and have certain recognition. Their names and related information are shown in Table 2. Among the six types of 3D printers, printers using the PPJ technique (Objet30 Pro and Projet 3510 HD Plus) utilize multiple print heads that extrude a micro-drop of photopolymer material onto a building platform and cure the material simultaneously; printers using the DLP technique (Perfactory DDP and DLP 800d) use a DLP projector controlled by a digital micromirror device to cure the photopolymer material in the entire layer with one exposure; and printers using the SLA technique (Form2 and Form3) use a rastered UV laser source directed by a scanner system to photocure all portions of a given slice in sequence.

**Figure 3:**
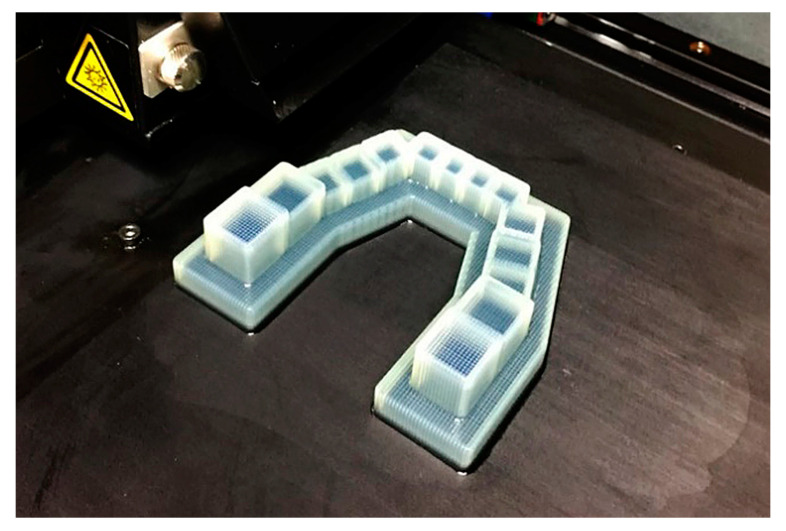
Model printing using the Objet30 Pro and a specific printing protocol.

The obtained 3D model was input into the data-processing software of each 3D printer and sliced into individual layers to be prepared for the building process. Considering the incremental layering pattern, to achieve a more targeted evaluation, a specific printing protocol was applied and is described as follows: ① the model base was placed on the printing platform, with the occlusal plane parallel to the platform (X-Y plane) and occlusogingival direction consistent with the *Z*-axis (Figure 3); ② the model was placed at the center of the printing platform to avoid possible illumination distortion at the edge of the platform; ③ the support-free printing mode and the printing parameters recommended by the manufacturer were selected, and the fully filled model was printed; and ④ post-processing according to the manufacturer’s instructions was strictly conducted.

One set of structurized dental models was printed by each printer. An optical scanner (Activity 880, Smartoptics, Munich, Bavaria, Germany) with an accuracy of 20 μm was used to scan each 3D printed model to acquire the digital data, which were output as an STL file. All models were measured immediately after printing and post-processing. The measurements, including the 3D form errors, occlusal plane errors, and occlusogingival direction errors, were performed by one trained examiner.

### 2.3. 3D Form Errors

All digital data were imported into the 3D analysis software Geomagic Studio 2013 (3D systems, Rockhill, SC, USA). The 3D form errors include the overall 3D deviation, flatness errors, parallelism errors, and perpendicularity errors. Considering that the distance between the mesial and distal surfaces of each SDC is close, this will affect the accuracy of 3D scanning. Therefore, in this study, when calculating the overall 3D deviation, the best-fit registration method of the Geomagic Studio 2013 software was used, and the data of the model base upper surface and the occlusal, buccal, and lingual surfaces of each SDC were selected to register the digital data of the 3D-printed model and original digital model. After registration, the deviation analysis module was used to calculate the 3D deviation root mean square (RMS) values of the selected area as the overall 3D deviation. The RMS values used to determine the averages of the positive and negative values were calculated using the following formula:

(1) $$RMS=\sqrt{\frac{{\sum_{i=1}^{n}\left(x_{1,i}-x_{2,i}\right)}^{2}}{n}}$$

where n is the sum of the measured points, x_1,i_ is the measurement point i on the reference digital model, and x_2,i_ is the measurement point i on the test digital model.

A color difference map with 21 color segments for each superimposition was generated to demonstrate the distribution of deviation. For color difference map construction, a maximum critical value of ±0.50 mm and nominal value of ±0.05 mm were set for the color spectra.

The data of the occlusal, buccal, and lingual surfaces of each SDC were selected to generate the corresponding best-fit planes—Plane O, Plane B, and Plane L via the best-fit method, respectively. The form deviation for each best-fit plane was recorded as a flatness error, which was calculated using the following formula:

(2) $$Flatness\;error=x_{positive}+\left.x_{negative}\right.$$

where x_positive_ is the distance of the farthest point on the positive side of the corresponding best-fit plane from that plane, and x_negative_ is the distance of the farthest point on the negative side of the corresponding best-fit plane from that plane.

The mean and standard deviation of 42 flatness error values were calculated, and the mean value was used as the flatness error for this model. The mean values of the flatness errors for 14 horizontal planes (Plane O) and 28 vertical planes (Plane B and Plane L) were also calculated separately.

**Figure 4:**
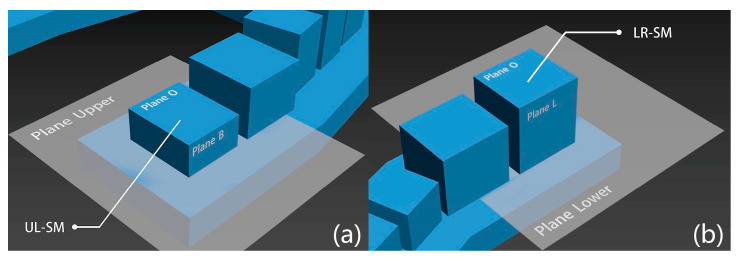
The generated corresponding best-fit planes using the best-fit method. (**a**) Best-fit plane of the upper surface of the base on the maxillary model (Plane Upper), best-fit planes of the occlusal surface and buccal surface of SDC UL-SM (Plane O and Plane B); (**b**) best-fit plane of the upper surface of the base on the mandibular model (Plane Lower), best-fit planes of the occlusal surface and lingual surface of SDC LR-SM (Plane O and Plane L).

The best-fit planes of the upper surface of the base on the maxillary and mandibular models were generated and defined as Plane Upper and Plane Lower (Figure 4). Different from the ISO definitions of parallelism errors and perpendicularity errors, this study redefined them in degrees, aiming to offer a more straightforward value for dentists to understand. The absolute value of the acute angle between each SDC’s horizontal plane and Plane Upper or Plane Lower was defined as the parallelism error. The angle between each SDC’s vertical plane and Plane Upper or Plane Lower was calculated and its absolute angle deviation from 90 degrees was defined as the perpendicularity error. The mean and standard deviation of 14 parallelism error values and 28 perpendicularity error values were calculated, and the mean values were used as the parallelism error and perpendicularity error for the model.

### 2.4. Linear Dimensional Error

**Figure 5:**
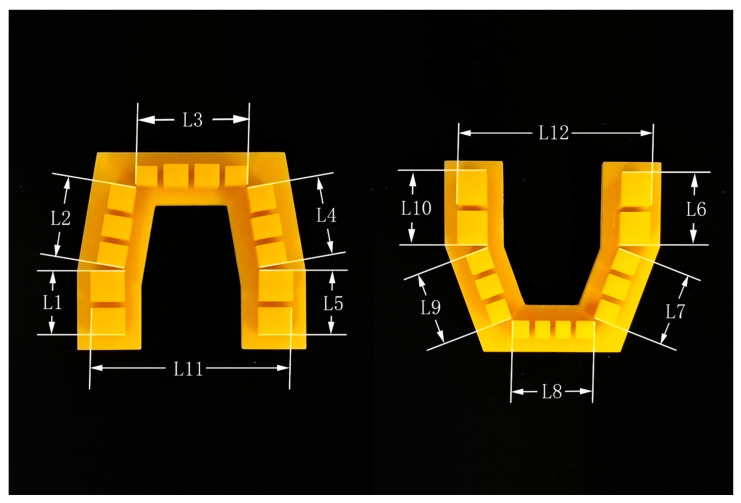
Linear measurement of the feature sizes of the simulated dental arches. The distance between: the distal surface of SDC UR-SM and mesial surface of SDC UR-FM (L1); the distal surface of SDC UR-SP and mesial surface of SDC UR-C (L2); the distal surface of SDC UR-LI and distal surface of SDC UL-LI (L3); the mesial surface of SDC UL-C and distal surface of SDC UL-SP (L4); the mesial surface of SDC UL-FM and distal surface of SDC UL-SM (L5); the distal surface of SDC LL-SM and mesial surface of SDC LL-FM (L6); the distal surface of SDC LL-SP and mesial surface of SDC LL-C (L7); the distal surface of SDC LL-LI and distal surface of SDC LR-LI (L8); the mesial surface of SDC LR-C distal surface of SDC LR-SP (L9); the mesial surface of SDC LR-FM distal surface of SDC LR-SM (L10); the buccal surface of SDC UR-SM and SDC UL-SM (L11); and the buccal surface of SDC LL-SM and SDC LR-SM (L12).

Linear measurements on the 3D-printed physical model were recorded using a hand-held digital caliper (SanLiang, Dongguan, Guangdong, China) with an accuracy of 10 μm. The caliper had narrow tip jaws that could be inserted into the interproximal areas of the printed model. The linear dimensions that were used to evaluate the occlusal plane error included the mesiodistal diameters (MDs) and buccolingual diameters (BLs) of each SDC, and the feature sizes of the simulated dental arches (L1–L12) (Figure 5), while the crown height (CH) of each SDC was used to assess the occlusogingival direction error.

All linear measurements were repeated five times, and the results were averaged. The relative error of the 96 measurement results was calculated using the following formula:

(3) $$Relative\;Error=\frac{x_{2}-x_{1}}{x_{1}}\times 100\%$$

where x_1_ is the designed dimension of the feature size on the reference digital model, and x_2_ is the measurement result of the feature size on the 3D-printed model.

The mean and standard deviation of 34 dimensions measured in the occlusal plane and 14 dimensions measured in the occlusogingival direction were calculated, and the mean values were used as the occlusal plane error and occlusogingival direction error, respectively. Occlusal plane error and occlusogingival direction error can also be regarded as printing errors in the printer’s X-Y plane and *Z*-axis, respectively.

The intra- and interexaminer reliability values of the repeated linear measurements were assessed using the intraclass correlation coefficient (ICC). To acquire the ICC values, examiner A made all linear measurements on one set of models three times successively. Examiner B and examiner C performed the same linear measurements immediately after examiner A.

## 3. Results

### 3.1. 3D Form Error

**Figure 6:**
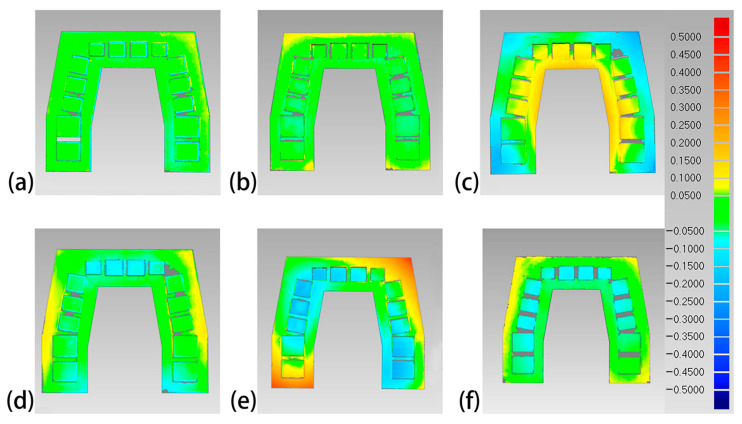
Occlusal view of the color difference maps of the printed maxillary models. (**a**) Objet30 pro; (**b**) Projet 3510 HD Plus; (**c**) Perfactory DDP; (**d**) DLP 800d; (**e**) Form2; and (**f**) Form3.

**Figure 7:**
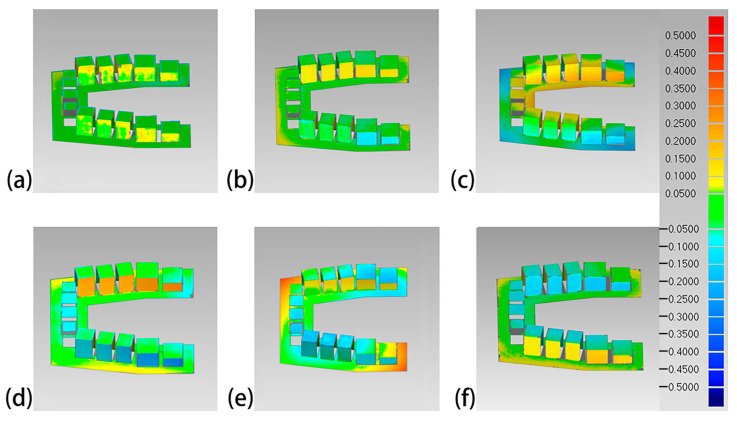
Lateral view of the color difference maps of the printed maxillary models. (**a**) Objet30 pro; (**b**) Projet 3510 HD Plus; (**c**) Perfactory DDP; (**d**) DLP 800d; (**e**) Form2; and (**f**) Form3.

**Figure 8:**
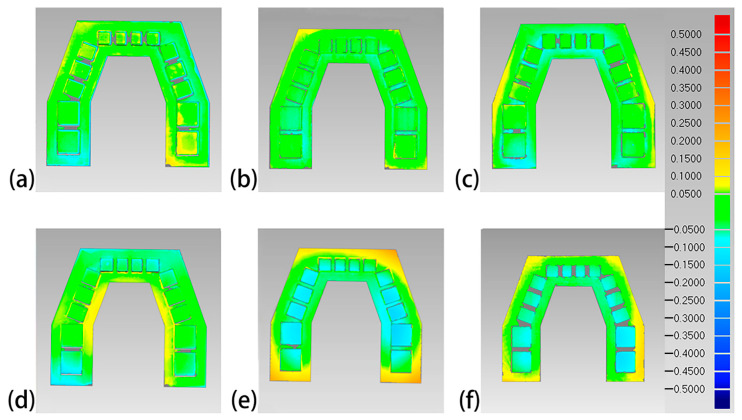
Occlusal view of the color difference map of the printed mandibular models. (**a**) Objet30 pro; (**b**) Projet 3510 HD Plus; (**c**) Perfactory DDP; (**d**) DLP 800d; (**e**) Form2; and (**f**) Form3.

**Figure 9:**
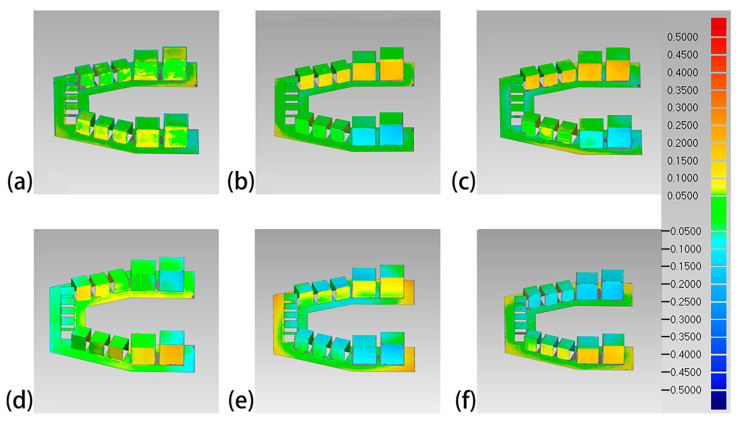
Lateral view of the color difference map of the printed mandibular models. (**a**) Objet30 pro; (**b**) Projet 3510 HD Plus; (**c**) Perfactory DDP; (**d**) DLP 800d; (**e**) Form2; and (**f**) Form3.

The overall 3D deviations of each set of 3D-printed models are shown in Table 3. The Objet30 Pro had the lowest overall 3D deviation, while the Perfactory DDP had the highest. The color difference maps are shown in Figure 6, Figure 7, Figure 8 and Figure 9. Expansion trends are depicted by the yellow color, while the blue color indicates contraction. From an occlusal view, warpage deformation trends were displayed on the model bases of the models printed by the Perfactory DDP, the DLP 800d, the Form2, and the Form3. From a lateral view, all models showed different degrees of transversal expansion or contraction trends on the buccal and lingual sides. The deviation of the posterior SDCs was more obvious than that of the anterior SDCs.

The flatness, parallelism, and perpendicularity errors of the six groups of printed models are shown in Table 4. With respect to the flatness performance, the smallest error of 0.061 ± 0.019 mm was found in the Form3-printed models, and the largest error of 0.190 ± 0.059 mm was found in Objet30 Pro-printed models. With regards to the parallelism performance, the smallest error of 0.138 ± 0.068° was found in the Objet30 Pro group, and the largest error of 0.453 ± 0.237° in the Form2 group. In terms of the perpendicularity performance, the Projet 3510 HD Plus printer with a perpendicularity error of 0.095 ± 0.070° was the best, and the Form2 printer with a perpendicularity error of 0.568 ± 0.183° was the poorest. The flatness errors on the horizontal and vertical surfaces of the six groups are presented in Table 5.

### 3.2. Linear Dimensional Error

**Figure 10:**
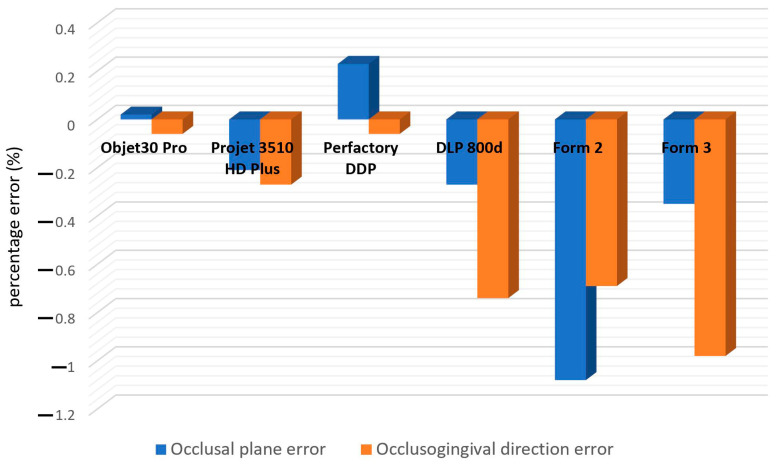
The occlusal plane error and occlusogingival direction error of the 3D printed models.

The intra- and inter-examiner ICCs for the linear measurements were 0.994 and 0.983, respectively. The linear measurement results of the six groups of printed models are shown in Table 6 and Figure 10. A positive value represents an enlargement of dimension, while a negative value represents a shrinkage. Dimension enlargement in the X-Y plane was observed in the Objet30 Pro and Perfactory DDP groups, while dimension shrinkage was observed in the other groups. Shrinkage along the *Z*-axis direction was observed in all groups. The Objet30 Pro group was the most accurate in terms of feature size.

## 4. Discussion

A recent systematic review conducted by Etemad-Shahidi et al. showed that the trueness of a 3D-printed dental model was greatly affected by the technical principles and the manufacturer of the specific device [11], which is also confirmed by our study. The six different test printers exhibited differing measurement results in terms of every 3D form error and linear measurement error. It is thought-provoking to find out that none of the printers were superior or inferior to the others in all aspects of spatial trueness, which proves the necessity of multi-dimensional evaluation.

The clinically acceptable range of errors established in previous studies was mostly 100 μm for prosthodontic and >250 μm for orthodontic models [31,43,44,45]. All test printers were deemed clinically acceptable in this respect.

### 4.1. Diversity in Spatial Trueness Evaluation Results of Different Printers

Owing to the development of proprietary software and desktop scanners, trueness evaluations can be easily conducted through the superimposition of a digital printed model and its master model. This 3D approach is highly automatic and considered to be less affected by manual errors, allowing for a more comprehensive and stable evaluation [46]. In the present study, the RMS value generated by the 3D analysis software(Geomagic Studio 2013) represents the overall 3D deviation of the printed models. The Objet30 Pro printer showed, significantly, the lowest overall 3D deviation among the groups. Camardella et al. used a 3D analysis method and found that the dental model with a horseshoe-shaped base printed by the Objet PPJ printer was more accurate than an SLA-device printed model, which is also in accordance with other studies [45,47]. Nevertheless, opposite conclusions seemed to be drawn by Favero et al. In their study, the Objet Eden260V printer showed the highest error in comparison with four other printers, even though its 3D deviation was under 100 μm, which is clinically acceptable [43]. This may have been due to the proprietary design of different Objet devices, and their designed printed model also being considerably different from ours. The Projet 3510 HD Plus, another printer using the PPJ technique, showed the second-lowest value of overall 3D deviation. This specific device was assessed by Jin et al., and the RMS value showed that the Projet PPJ printer has a better trueness than the FDM printer when printing a full-arch dental model and has no significant difference compared with traditional plaster models [14]. In another study by Jin et al., the 3D trueness of the Projet PPJ printer was inferior to the Projet SLA printer. This was explained by the fact that the SLA model had less polymerization shrinkage and material evaporation because of faster multiple polymer photo-curing than the PPJ model [20]. Recently, DLP printers have received increasing interest in respect to rapid prototyping for their potential in high-speed reconstruction. A wide selection of DLP printers have come under the spotlight. The Perfactory DLP printer, one of the mainstream choices, was assessed in our study. Its overall 3D deviation turned out to be the highest among the groups, yet still below the 100 μm threshold. Mangano et al. concluded that the Perfactory DLP printer exhibited a superior 3D trueness compared to five other desktop printers and a similar conclusion was also drawn by Favero et al. [43,48]. However, all models were assessed one month after printing in a study by Mangano et al., leading to dimensional changes caused by aging [49]. Furthermore, the information on the printing material used was not clearly identified in either study [50]. The DLP 800d printer was assessed first, to our knowledge, and its overall 3D deviation was slightly lower than the Perfactory printer. Using a 3D analysis method, the trueness performance often varies between different printers using the very same technique, and the same goes for SLA printers [11]. Two Form SLA printers were assessed, and both printers showed an overall 3D deviation under 100 μm, while the Form3 printer was somewhat better than its predecessor. Past studies have proved that the Form2 SLA printer is capable of printing accurate models with clinical acceptable 3D deviation [43,44,46], making it reasonable to infer the comparable reliability of the Form3.

Although 3D comparison followed by surface-based superimposition can well identify overall shape differences, it is also important to note that the amount of deviation is rarely distributed evenly, as shown in the color difference maps (Figure 6, Figure 7, Figure 8 and Figure 9). The models printed with resin matrix composites have the characteristics of shrinkage and warpage [2,12,30], and the warpage on the entire dental model becomes a bottleneck in the 3D comparison. Linear measurement still plays an irreplaceable role in evaluating the regional differences of 3D-printed models. In our study, the Objet30 Pro printer showed the lowest percentage error in the linear measurement of feature sizes (0.02% in the occlusal plane and 0.06% in the occlusogingival direction). The highest linear deviation was found in the Form2 and Form3 groups. An array of previous studies has assessed the trueness of 3D-printed dental models by linear measurements. The design of the test model, definition of feature sizes, and evaluation tools differed, making the measured values themselves not comparable. According to Brown et al., the Objet PPJ printer had a better linear trueness than the DLP printer, especially for crown height measurements [17]. Similarly, Camardella et al. found that printed dental models using the Objet PPJ printer have a lower linear error than SLA printers, and these findings were further supported by other studies [27,28,30]. Nestler et al. compared the linear measurement results of DLP and SLA printers, and the DLP printers showed a better consistency with the master file, in agreement with our results [51]. When compared to traditional plaster models, Hazeveld et al. concluded that the Objet PPJ printer and EnvisionTEC DLP printer both have low mean systematic differences [26]. In our study, the highest linear percentage error was 1.08% for the Form2 printer, which means a 108 μm discrepancy when printing a ten-millimeter object. In this regard, this device is still suitable for clinical use, especially for orthodontics applications. In addition, Mangano et al. proved that the Form2 printer was the most accurate device in linear measurements among six printers when printing small-size objects [48].

The main reason accounting for the diversity in the spatial trueness of printers is related to the different formation principles of these photopolymerization-based printing techniques. The PPJ technique utilizes multiple print heads that extrude a micro-drop of photopolymer material onto a building platform and cure the material simultaneously [52]. This markedly reduces the deformation caused by extensive polymerization shrinkage, making the overall and local shape accurate [2]. Another widely applied technique is DLP, which uses a DLP projector controlled by a digital micromirror device to cure the photopolymer material in the entire layer with one exposure and achieve faster production. Using this building principle, the DLP printer has a relative advantage when forming a flat surface. The models printed by two DLP printers in our study showed the lowest flatness error and acceptable parallelism and perpendicularity errors. However, massive curing within each layer increases shrinkage stress, making the overall curl distortion of the material more evident. Similar to DLP printers, the SLA printer also showed an excellent flatness error and relatively poor overall trueness, with the largest warpage deformation trend observed in the color difference maps. For the SLA technique, a rastered UV laser source directed by a scanner system photocures all portions of a given slice in sequence, in a vat of photopolymer resin [52,53]. Increased shrinkage stress also occurs on SLA-printed models [13].

From what has been discussed above, attention should be paid to the trueness of every kind of 3D printer using both the 3D analysis and linear measurement methods. A 3D analysis can effectively show the overall model deformation caused by warpage, while the linear measurement quantifies the linear deviation, which has a better reference value for dentists. In our study, the Perfactory DDP printer showed a good linear trueness, but its overall 3D deviation was the highest among the groups. Taking both aspects into consideration, the structurized dental model printed by the Objet30 Pro printer had the best trueness.

### 4.2. Feasibility of Multi-Dimensional View of Spatial Trueness Offered by the Structurized Dental Model

As previously stated, the 3D analysis method only offers an overall value of the entire surface changes, and superimposition is suitable only to a limited extent for assessing dimensional deviations [51]. On the other hand, the lack of reproducible measuring points on the irregular surface leads to a certain degree of measurement errors in manual linear measurements [4]. Establishing a geometric reference model with a well-designed shape can efficiently overcome this problem, since it is able to simplify landmark selection. Reference models have been used in assessing the accuracy of 3D-printed single-unit prostheses, multi-unit prostheses, and dental models [36,48,54,55,56,57]. In the ISO standard (ISO 23298:2023) [58], geometric reference models simulating different prostheses were used to test the machining accuracy of milling machines. A similar method to ours, which applies a geometric model simulating a full-arch dental model, was proposed by Emir and Ayyildiz [56]. In this study, a model with a horseshoe-shaped base and six cylindrical abutments was established. However, this reference model did not carefully refer to the dimensions of clinical dental models or manifest the size differences between teeth. To the best of our knowledge, the structurized dental model proposed by our study is the first reference model simulating the shape of a clinical full-arch dental model using geometric structures, allowing for planar as opposed to point-to-point measurements, therefore reducing the difficulty in making linear measurements using calipers. However, when calculating the overall 3D deviation, we used a high-precision optical scanner (Activity 880, with an accuracy of 20 μm) to obtain the digital data of the printed model. Although the same optical scanner was used, there were still inevitable scanning errors, which will affect the results of this study to some extent. [56]. But according to the evaluation results of printing accuracy in this study, we think that scanning errors had little influence on the research results.

According to the ISO standard (ISO 1101:2017) [59], flatness, parallelism, and perpendicularity are important types of geometrical tolerance. Flatness, parallelism, and perpendicularity errors have already been used in previous studies to evaluate the shape accuracy of 3D-printed parts [60,61]. Whatever kind of printing technique used, the curing process of a photo-curing 3D printer is repeated layer-by-layer. The basic building block of printed parts is a thin layer with a specific thickness, and this is fundamental for trueness evaluation. Additionally, the shoulder area of a 3D-printed abutment preparation has a shape highly resembling a flat surface [62]. In our study, all tested flat surfaces were either parallel or perpendicular to the building platform. In this way, the flatness, parallelism, and perpendicularity errors can be easily assessed in 3D analysis software(Geomagic Studio 2013), directly demonstrating the quality of layer production (Table 3). The flatness error of vertical planes can also reflect the surface roughness, which is particularly affected by stacking pitch [63].

Although inaccurate modeling related to polymerized-induced shrinkage occurs in both the horizontal and vertical directions of photo-curing 3D printers, the former is more relevant to the resolution of the light source (pixel size or spot size) and the printer’s ability to accurately locate the irradiation exposure, while the latter is often associated with the depth of cure, chemical bonding, and layer thickness setting [52,64,65]. Considering the placement strategy of a model when printing, the CH of a cuboid simulated dental crown can reflect the dimensional trueness in the vertical direction, while other feature sizes can represent the horizontal direction. As depicted in Figure 10 and Table 5, the trueness in these two directions is always different, and it is necessary to offer both lots of information to practitioners in order to choose their suited printing orientation [36].

The meticulous and detailed measurement of this trueness evaluation method using a structurized model is challenging for printers. Printers that exhibit an excellent trueness in this rigorous method should be dependable and eligible for varying clinical scenarios. Generally, one 3D printer is used for multiple purposes, especially in private practice. This evaluation result measured from the structurized dental model can provide a reference for the trueness of most 3D-printed models with dental arch characteristics, such as prosthodontic working casts, orthodontic diagnostic casts, and even implant surgical guides.

### 4.3. Future Works to Optimize the Evaluation Process

The evaluation of accuracy involves two aspects: trueness and precision. The precision of a 3D-printed dental model refers to the repeatability of printing the same model multiple times. Under the same printing conditions, most current mainstream 3D printers have a relatively good precision with proper operation [14,20]. Trueness, on the other hand, refers to the consistency of the shape and size of a printed model with its true value. Compared with precision, which can be influenced by the printing environment and manual operation, trueness is more dependent on the principles and property of the printing equipment and raw material used, which was the main focus of this study.

The properties of the printing material are the main causes of model deformation. At present, most printing materials use epoxy compounds and acrylic compounds at the same time to initiate photopolymerization with a mixed curing system, so as to achieve better accuracy and mechanical properties [66]. Utilizing the ring-opening polymerization reaction of some epoxy compounds, prepolymers with larger molecular weights, expandable fillers, and monomers can avoid the curing shrinkage of printing materials to a certain extent. However, considering factors such as solvent volatilization and the poor controllability of the curing degree of the material during the molding process, material shrinkage cannot be completely avoided [67], thus affecting the accuracy of 3D printing. Factors such as the illumination intensity, wavelength, and exposure time of the printer will also affect the accuracy of 3D printing. The parameters of the molding layer thickness will especially affect the accuracy in the *Z*-axis direction. When the layer thickness parameter is too large, the adhesion between adjacent thin layers may be insufficient, resulting in local peeling of the model and degradation of its mechanical properties; when the layer thickness parameter is too small, the *Z*-axis error will increase with an increase in the number of layers [68]. The optimum printing materials and printing parameters of different printers are not consistent. In order to reflect the best printing performance of different printers and make a better horizontal comparison, the printing materials and printing parameters recommended by manufacturers were selected in this study. In this study, the influences of printing materials and equipment on printing accuracy were not evaluated separately, but the method of this study can be used to evaluate the printing accuracy of the same printer using different printing materials, as well as the printing accuracy of different printers using the same printing materials. Related research needs to be carried out in the future.

Precision evaluation using this structurized dental model will be the topic of our future study. The printing accuracy of a model is one of the important indicators for evaluating the performances of printers at present, but besides a high accuracy, dental models also need to have characteristics such as a good biological safety, high strength, and good aesthetics, etc., which is also the focus of our future study.

Aside from the printing techniques and manufacturing material, it has been reported that many other factors can have an effect on the accuracy of 3D-printed dental models, such as the manufacturing parameters [32,43,46,69], placement position on the build plate [32,48], printing direction [36,70,71], internal and external structure design [9], number of support structures [69], postprocessing protocol [72], sterilization process [73], and aging of the material [49,74]. We believe this standardized trueness assessment protocol will smooth the way for further investigating the influence of the aforementioned factors and can be used to evaluate 3D printers’ performance and different printing techniques.

This study also has some limitations, as follows: ① compared with real tooth shapes, the shape of the SDC constructed in this study was relatively abstract; ② in order to provide a better view of the 3D printer’s performance on reduction in the transverse dimension, the maxillary model in this study had no palatal structure; and ③ in this study, a support-free printing mode was adopted, which has limited guiding significance for the clinical application of supported printing. In view of the above limitations, corresponding improvements will be made in the future study.

## 5. Conclusions

Within the limitations of this in vitro study, the following conclusions were drawn:

① The overall 3D deviations of the six types of photo-curing 3D printers ranged from 45 μm to 95 μm. It can be inferred that all the dental models printed by the test printers were considered as clinically acceptable for orthodontics and prosthodontics, etc.

② According to the evaluation results, the Objet30 Pro printer had the best spatial trueness when taking both the 3D analysis and linear measurement methods into consideration.

③ The method proposed in this study can objectively and comprehensively evaluate the spatial trueness of 3D-printed dental models made of photopolymer resin and has a good repeatability and generalizability.

## Figures and Tables

**Table 1:** Dimensions of the simulated dental crowns (SDC) of the structurized dental model (unit: mm).

SDC	Maxillary Model			Mandibular Model		
	Mesiodistal Diameter	Buccolingual Diameter	Crown Height	Mesiodistal Diameter	Buccolingual Diameter	Crown Height
Central incisor	8	7	10	5	6	8
Lateral incisor	7	6	9	6	6	8
Canine	8	8	11	7	7	8
First premolar	7	9	10	7	8	7
Second premolar	7	9	10	7	8	7
First molar	10	11	8	11	10	10
Second molar	9	11	5	11	10	12

**Table 2:** Names and related information of six types of photo-curing 3D printers.

3D Printer Name	Manufacturer	Printing Technology	Printing Materials	Layer Thickness (μm)	XY Resolution (μm)
Objet30 Pro	Stratasys (Eden Grasslands, MN, USA)	Polyjet	VeroClear light-curing resin	16	42
Projet 3510 HD Plus	3D systems (Rockhill, SC, USA)	Multijet	VisiJet EX200 light-curing resin	16	34
Perfactory DDP	EnvisionTEC (Mar, Ruhr, Germany)	DLP	E-model light light-curing resin	50	60
DLP 800d	Han’s laser (Shenzhen, Guangdong, China)	DLP	T-MOG-522 light-curing resin	50	75
Form2	Formlabs (Somerville, MA, USA)	SLA	Dental Model light-curing resin	50	140
Form3	Formlabs (Somerville, MA, USA)	SLA	Dental Model light-curing resin	50	85

**Table 3:** The overall 3D deviation of the 3D-printed models (unit: μm).

	Overall 3D Deviation		
	Maxillary Model	Mandibular Model	Overall Value
Objet30 Pro	47	43	45
Projet 3510 HD Plus	55	72	64
Perfactory DDP	97	92	95
DLP 800d	98	72	85
Form2	102	79	91
Form3	74	87	80

**Table 4:** The flatness, parallelism, and perpendicularity errors of the 3D-printed models.

	Flatness Error (mm)	Parallelism Error (°)	Perpendicularity Error (°)
Objet30 Pro	0.190 ± 0.059	0.138 ± 0.068	0.265 ± 0.191
Projet 3510 HD Plus	0.081 ± 0.024	0.141 ± 0.071	0.095 ± 0.070
Perfactory DDP	0.074 ± 0.023	0.434 ± 0.244	0.478 ± 0.273
DLP 800d	0.075 ± 0.019	0.262 ± 0.090	0.214 ± 0.161
Form2	0.076 ± 0.020	0.453 ± 0.237	0.568 ± 0.183
Form3	0.061 ± 0.019	0.270 ± 0.094	0.151 ± 0.122

**Table 5:** The average flatness error of the horizontal planes and vertical planes of the 3D-printed models (unit: mm).

	Flatness Error of Horizontal Planes	Flatness Error of Vertical Planes
Objet30 Pro	0.250	0.161
Projet 3510 HD Plus	0.081	0.081
Perfactory DDP	0.056	0.083
DLP 800d	0.068	0.079
Form2	0.065	0.081
Form3	0.042	0.071

**Table 6:** The occlusal plane errors and occlusogingival direction errors of the 3D-printed models (unit: %).

	Occlusal Plane Error	Occlusogingival Direction Error
Objet30 Pro	0.02 ± 0.36 *	−0.06 ± 0.09
Projet 3510 HD Plus	−0.21 ± 0.30	−0.27 ± 0.14
Perfactory DDP	0.23 ± 0.36	−0.06 ± 0.15
DLP 800d	−0.27 ± 0.24	−0.74 ± 0.11
Form2	−1.08 ± 0.38	−0.69 ± 0.19
Form3	−0.35 ± 0.15	−0.98 ± 0.14

* 0.02% is equivalent to a 2 μm discrepancy for a 10 mm printed object.

## Data Availability

The datasets used and analyzed during the current study are available from the corresponding author on reasonable request.
